# Comparative Metabolomic Analysis of the Neuroprotective Effects of Scutellarin and Scutellarein against Ischemic Insult

**DOI:** 10.1371/journal.pone.0131569

**Published:** 2015-07-06

**Authors:** Hao Tang, Yuping Tang, Nian-Guang Li, Hang Lin, Weixia Li, Qianping Shi, Wei Zhang, Pengxuan Zhang, Zexi Dong, Minzhe Shen, Ting Gu, Jin-Ao Duan

**Affiliations:** Jiangsu Collaborative Innovation Center of Chinese Medicinal Resources Industrialization, Jiangsu Key Laboratory for High Technology Research of TCM Formulae, National and Local Collaborative Engineering Center of Chinese Medicinal Resources Industrialization and Formulae Innovative Medicine, Nanjing University of Chinese Medicine, Nanjing, Jiangsu, 210023, China; University of Kansas, UNITED STATES

## Abstract

For more than thirty years, scutellarin (Scu) has been used in China to clinically treat acute cerebral infarction and paralysis. Scutellarein (Scue), the major Scu metabolite in vivo, exhibits heightened neuroprotective effects when compared to Scu. To explore the neuroprotective role of these compounds, we performed ultra-high-performance liquid chromatography-quadrupole/time-of-flight mass spectrometry (UHPLC-QTOF/MS) coupled with a pattern recognition approach to investigate metabolomic differences in a rat model of ischemia after treatment with each compound. We examined metabolites in urine, hippocampal tissue, and plasma, and we tentatively identified 23 endogenous metabolites whose levels differed significantly between sham-operated and model groups. Upon pathway analysis, we found an additional 11 metabolic pathways in urine, 14 metabolic pathways in the hippocampal tissue, and 3 metabolic pathways in plasma. These endogenous metabolites were mainly involved in sphingolipid metabolism, lysine biosynthesis, and alanine, aspartate, and glutamate metabolism. We found that metabolic changes after ischemic injury returned to near-normal levels after Scue intervention, unlike Scu treatment, further validating the heightened protective effects exerted by Scue compared to Scu. These results demonstrate that Scue is a potential drug for treatment of ischemic insult.

## Introduction

Stroke remains one of the major causes of death and severe disability worldwide [1]. There are two major types of stroke which are ischemic and hemorrhagic, and ischemic stroke is the most common, and they occur when an artery is blocked by a blood clot. This usually results in insufficient blood supply to one or more cerebral vessels followed by a restoration in blood flow to the cerebral tissue, further exacerbating cerebral damage due to reperfusion injury [2]. Bioenergetic failure, oxidative stress, inflammation, activation of platelet-activating factor, and cell death after acute cerebral ischemia hypoperfusion all contribute to neural injury following ischemic stroke [3].

Currently, there are two major therapeutic approaches to treat acute ischemic stroke. The first approach relies on some neuroprotective agents to target biochemical pathways, regulating cell fate in order to protect cerebral function and enhance neuronal repair and recovery. However, neuroprotective agents show weak efficacy, and they are limited by side effects. The second approach involves the administration of thrombolytic drugs, within a 3-hour treatment window post-ischemic stroke, to restore blood flow to the brain. While these drugs exhibit positive effects for acute ischemic strokes if they are administered within the treatment window, they also have serious side effects, such as hemorrhage [4, 5].

**Fig 1:**
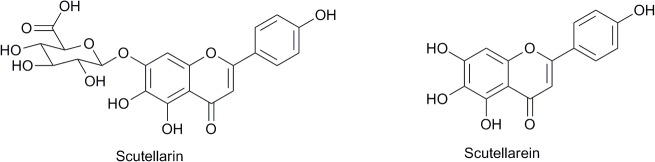
The chemical structures of scutellarin and scutellarein.

Scutellarin (Scu) (Fig 1) is the main active compound in breviscapine, which is extracted from the Chinese herb *Erigeron breviscapus* (vant.) Hand. Mazz. This herb has been used clinically to treat acute cerebral infarction and paralysis induced by cerebrovascular diseases such as hypertension, cerebral thrombosis, and cerebral hemorrhage in China since 1984 [6]. Modern pharmacological studies have demonstrated that Scu treatment induces neuroprotective effects, by acting as a vasodilator, antioxidant, and anti-inflammatory agent. Scu also plays a role in anti-excitotoxicity and the blockage of calcium (Ca^2+^) channels [7–12]. Within the intestine, Scu is more readily absorbed in its hydrolyzed form, scutellarein (Scue) (Fig 1). One study reported that Scue was more easily absorbed after oral administration than Scu, when administered in equal doses. Previous studies have also demonstrated that Scu and Scue could prevent neuronal injury and Scue have better protective effects than Scu in a rat cerebral ischemia model [13]. However, the precise mechanisms mediating the neuroprotective role of Scu and Scue remain unknown.

Metabolomics is an ideal approach for assessing the overall physiological status of an organism by comprehensive analysis of metabolic changes occurring in response to genetic, environmental, or lifestyle factors [14, 15]. Furthermore, evidence of drug efficacy can be also obtained from detailed metabolomics analyses showing changes in low molecular weight metabolites [16]. Disease pathogenesis and the cellular mechanisms affected by drug therapies may be clarified through analysis of disease biomarkers. Biomarkers also are useful for diagnosing and monitoring disease progression, and they are important to consider when selecting the appropriate treatment for a patient as well as monitoring side effects [17, 18].

Various analytical techniques, such as UHPLC-TOF/MS, gas chromatography-mass spectrometry (GC-MS) and nuclear magnetic resonance (NMR), have been applied in drug metabolic studies. UHPLC-QTOF/MS coupled with multivariate data analysis techniques has proven effective for biomarker discovery [19–27]. Therefore, we used a metabolomics approach, based on the UHPLC-QTOF/MS technique, coupled with pattern recognition to investigate the metabolomic profiles in order to identify biomarker expression in an ischemia model after treatment with Scu or Scue. Metabolomic pathways in the ischemia model were constructed using the Metabolomics Pathway Analysis (MetPA) online database to determine the metabolic characteristics of urine, plasma, and hippocampal tissue and to compare the metabolic differences between treatment with Scu and Scue.

## Materials and Methods

### Chemicals and reagents

Scu was purchased from Mianning Jiexiang Co. Ltd. (Chengdu, China). Scue was prepared according to our previously reported methods [28, 29]. Nimodipine was purchased from Central Pharmaceutical Co. Ltd. (Tianjin, China). Acetonitrile and methanol were of HPLC grade and purchased from Tedia (Fairfield, OH, USA). Analytical grade formic acid was purchased from Merck (Darmstadt, Germany). Deionized water was purified using a Millipore water purification system (Millipore, Milford, MA, USA) and filtered with 0.22-μm membranes. All other reagents used were of analytical grade. l-Aspartic acid, l-citrulline, and lysoPC (18:0) were obtained from Sigma-Aldrich (St. Louis, MO, USA).

### Animals

Male Wistar rats (5 to 6 weeks, 180g) were provided by the Shanghai Slac Laboratory Animal Co. Ltd. (Shanghai, China). The rats were raised to about 240g, then an initial acclimation period of 3 days in cages, the rats were transferred to metabolic cages and allowed to acclimatize for 3 days on a 12 h dark-light cycle at a temperature of 23 ± 2°C with a humidity of 50 ± 5%; they were allowed free access to food and water. The protocol was approved by the Animal Experimental Ethical Committee of Nanjing University of Chinese Medicine. All efforts were made to ameliorate animal suffering [30].

### Animal treatment and sample collection

After one week of acclimation, 40 rats were randomly divided into 5 groups of 8 rats including: 1) Surgery without bilateral common carotid artery occlusion (sham-operated group); 2) Bilateral common carotid artery occlusion (BCCAO) rats without pretreatment (model group); 3) BCCAO rats pretreated with nimodipine (0.03 mmol/kg) (positive control group); 4) BCCAO rats pretreated with Scu (0.35 mmol/kg); and 5) BCCAO rats pretreated with Scue (0.35 mmol/kg). The rats in the pretreated groups were administered Scu or Scue intragastrically at a dose based on body weight for 6 consecutive days, while the rats in the sham-operated and model groups were orally administered an equivalent volume of 0.25% sodium carboxymethyl cellulose (CMC-Na).

The brain was sensitive to temperature variations during the anesthesia interval and early recovery. Lowering the body temperature of the rats is likely to result in slowly evolving neuropathology, and more seriously, it may result in death in rats. Rats were anesthetized with 10% chloral hydrate (350 mg/kg) via intraperitoneal (IP) injection on the 7th day. Each anesthetized rat was placed on 37.0°C temperature-controlled pad. The arteries of BCCAO rats were exposed and ligated for 30 min to induce cerebral ischemia, and cerebral ischemia-reperfusion was achieved by de-clamping the arteries for 15 min. Then, the ischemia was applied for an additional 30 min and reperfusion was allowed to occur for 22 h. Anesthetized rats were placed on a heating pad during recovery from anesthesia to maintain their body temperature at 37.0 ± 0.5°C. During the surgery period, every rat’s temperature was monitored continuously by measuring anal temperature, and excluded the rats that the body temperature changed largely. The sham-operated rats were subjected to a bilateral common carotid isolation without occlusion. The bilateral common carotid artery occlusion model was easier to perform, and the less-intrusive surgical intervention allows greater scope for recovery experiments, Besides, we examined the response of the rat brain to secondary ischemia insult to produce hippocampal cell death via pre-experiment, and found that ischemia for 30 min and reperfusion for 15 min, then ischemia for 30 min and reperfusion for 22 h exhibited cell death in the hippocampal CA1 area [13, 31–33].

After 22 h of reperfusion, the rats were housed in metabolic cages and were allowed to free access to a standard diet and water. Rat urine samples were collected on the 8th day. The prior 0–12 h was labeled sample 1, and the following 12–24 h was labeled sample 2. Samples 3, 4, 5, and 6 were collected on the 9th and 10th days (1 sample every 12 h). The urine samples were immediately centrifuged at 3000 r for 10 min after collection. The urine supernatant was separated and stored at –80°C until analysis. In the last day, blood samples (0.7 mL) were collected from the posterior orbital venous plexus; each blood sample was centrifuged at 3000 r for 10 min to obtain plasma that was then stored at –80°C until analysis. The rats were deeply anesthetized and sacrificed. The whole cerebrum was removed from the skull on ice, and the hippocampal tissue was carefully isolated. All brain tissue samples were stored at –80°C until analysis.

### Biochemical indicator measurements

Therapeutic efficacy of Scu and Scue treatment was evaluated by analyzing superoxide dismutase (SOD) levels, the brain index, brain water content, Na^+^/K^+^-ATPase and Ca^2+^-ATPase activity, as well as malondialdehyde (MDA), sodium (Na^+^), potassium (K^+^), and calcium (Ca^2+^) concentrations in hippocampal tissue. Biochemical analysis data are expressed as the mean ± SD. Group differences were assessed using an analysis of variance (ANOVA) test. A value of *p* < 0.05 was considered statistically significant.

### Sample preparation

Prior to analysis, urine samples were thawed at room temperature and centrifuged at 13000 r for 10 min. Urine supernatant (0.5 mL) was added to 0.5 mL of acetonitrile and vortexed for 2 min and centrifuged again at 13000 r for 10 min to obtain the supernatant. Plasma (0.2 mL) was added to 0.6 mL of acetonitrile, vortexed for 2 min and then centrifuged at 13000 r for 10 min to obtain 0.6 mL of supernatant. Hippocampal tissues (0.1 g) were homogenized in 1.5 mL cold deionized water. Tissue samples were then centrifuged at 3000 r for 10 min at 4°C. The upper layer (1.2 mL) was transferred into another tube and 3 volumes of acetonitrile was added. The resulting mixture was vortexed for 2 min and centrifuged at 13000 r for 10 min. The supernatants from the plasma and hippocampal tissue were dried using a rotary evaporator at 25°C, and then the residues were re-dissolved in 200 μl of 50% acetonitrile in water by vortexing for 3 min. After centrifugation at 13000 r for 15 min at 4°C, the supernatants were transferred to auto-sampler vials for metabolomic analysis.

To validate the stability of the liquid chromatography-mass spectrometry (LC–MS) system, we randomly selected either plasma, urine, or hippocampal tissue samples from each group as quality control (QC) samples, and they were mixed together, respectively. This pooled tissue provided a representative “mean” sample containing all analytes that were encountered during the analysis. Moreover, to condition or equilibrate the system, the QC samples were injected five times at the beginning of the run and then every ten samples to further monitor the stability of the analysis. The QC data obtained were used to investigate the analytical variability in the whole run. This was necessary to evaluate whether the analytical system had changed (and to what extent) over the course of the analysis; moreover, this was essential for evaluating the variation in the analytical results, and therefore the reliability of the metabolite profiling data [30].

### UHPLC-QTOF/MS analysis conditions

Samples were run in a random order, both in group and sample type. The UHPLC analysis was performed on a Waters ACQUITY UPLC system (Waters Corporation, Milford, MA). An Acquity UPLC BEH-C_18_ column (2.1 mm × 50 mm, 1.7 μm) was used for all analyses. The column was maintained at 40°C, and a gradient of 0.1% formic acid in water (solvent A) and acetonitrile (solvent B) was used. The gradient used for urine samples was as follows: 3.0%–35% B over 0–10.0 min; 35%–80% B over 10.0–12.0 min; and 80% for 2.0 min, after which it was returned to 3% B for 2.0 min. The gradient used for plasma samples was as follows: 5.0%–35% B over 0–2.0 min; 35%–75% B over 2.0–8.0 min; 75%–95% B over 8.0–12.0 min; and 95% for 1.0 min, after which it was returned to 5% for 2.0 min. The gradient used for hippocampal tissue samples was as follows: 3.0%–80% B over 0–3.0 min; 80%–95% B over 3.0–12.0 min; and 95% for 1.0 min, after which it was returned to 3% for 2.0 min. The flow rate was set to 0.4 mL/min, and a 5-μl aliquot of the sample solution was injected into the BEH-C_18_ column.

Mass spectrometry was performed on a SYNAPT Q-TOF mass spectrometer (Waters, Manchester, U.K.). UHPLC-QTOF-MS was used for the relative quantification analysis of potential biomarkers. The parameters in the source were set as follows: capillary voltage, 3.0 kV; source temperature, 150°C; desolvation temperature, 550°C; cone gas flow, 50 L/h; and desolvation gas flow, 1000 L/h. Data were acquired between a m/z of 50 and 1000 Da with a 0.3 s scan time and a 0.1 s interscan delay. Data were processed further using MassLynx 4.1 software (Waters). Leucine-enkephalin was used as the lock mass, generating an [M + H]^+^ ion (*m/z* 556.2271) and [M–H]^−^ ion (*m/z* 554.2615) at a concentration of 200 pg/mL and a flow rate of 100 μl/min to ensure accuracy during the MS analysis via a syringe pump.

### Data processing and multivariate data analysis

All raw mass spectrometric data for urine, plasma, and hippocampal tissues were processed using MassLynx V4.1 and MarkerLynx software (Waters Corp., Milford, MA). UHPLC-MS data detection and noise-reduction was performed in both UHPLC and MS domains so that only true analytical peaks remained upon software processing. The ion intensity was normalized with respect to the total ion count (TIC) to generate a data matrix including the retention time, *m/z* value, and the normalized peak area. The mass data acquired were analyzed using EZinfo 2.0 software (Waters Corp, Milford, USA) for peak detection and alignment [19]. The main parameters of the MarkerLynx method were set as follows: mass tolerance 0.1 Da; noise elimination level 6; full scan mode employed in the mass range of 50–1000 amu; the initial and final retention times set to 0.5 and 14.5 min for data collection.

Principal components analysis (PCA) is an unsupervised analysis approach, and it was used to convert multiple original variable spaces into a new set of orthogonal variables. The supervised partial least-squared discriminate analysis (PLS-DA) and orthogonal partial least-squared discriminate analysis (OPLS-DA) were applied to determine the various metabolites responsible for the separation between the model and sham-operated groups. The variable importance in the projection (VIP) values, a weighted sum of squares, was used to select biomarkers. Variables with a VIP value greater than 1 demonstrated a higher influence on classification [34]. The goodness of fit and the evaluation of the OPLS-DA model were evaluated using the model parameters R^2^Y, representing the fraction of explained Y-variation, and Q^2^, the estimated predictive ability [19]. Ultimately, the differential metabolic features associated with the model and Scu or Scue treatment groups were obtained in accordance with the cutoff levels based on both VIP values and critical *p*-values from univariate analysis.

Using SPSS v18.0, differences between the Scu-treated, Scue-treated, nimodipine-treated, sham-operated group, and model groups were analyzed using an ANOVA test, assuming non-normal data distribution; *p*-values < 0.05 were considered significant [35].

### Construction of metabolic pathways

The metabolic pathways of endogenous metabolites could be constructed using MetPA (http://metpa.metabolomics.ca/MetPA/faces/Home.jsp), based on database sources, including Kyoto Encyclopedia of Genes and Genomes **(**KEGG) (http://www.kegg.jp/), Human Metabolome Database (HMDB) (http://www.hmdb.ca/), and Small Molecule Pathway Database (SMPD) (http://www.smpdb.ca/), to identify, analyze, and visualize metabolic pathways affected in treatment groups.

## Results and Discussion

### Biochemical Analysis

The mortality rates were showed in the Table 1. The rats in the sham-operated and Scue group did not die, there were one dead rat in the Model and Nimodipine group, respectively, but there were two dead rats in Scu group. The cause of death may be serious ischemia and hypothermia. The metabolite indicator levels detected in the model group were significantly altered in comparison to the group (*p* < 0.01). However, after oral administration of Scu, Scue, and nimodipine, the indicators were restored to near sham-operated levels. Oxidative stress acts to balance pro-oxidants and antioxidants. Scu and Scue significantly reduced the concentration of MDA and increased the activity of SOD (*p* < 0.01). Ischemic cerebral edema is a predominant clinical feature in global cerebral ischemia/reperfusion injury. Pretreatment with Scu and Scue produced a significant reduction in the water content. Furthermore, pretreatment of Scu and Scue significantly prevented increase in Na^+^ and decrease in K^+^ levels by protecting against a reduction in Na^+^/K^+^-ATPase activity and by causing a lower net ion shift, resulting in reduced cerebral edema. An overload of intracellular Ca^2+^ may be related to the pathophysiology of cerebral ischemia. Scu and Scue significantly decreased the level of Ca^2+^ and increased Ca^2+^-ATPase activity (*p* < 0.05 and *p* < 0.01, respectively) (Table 1). These results indicate that Scu and Scue can attenuate neuronal injury by regulating multiple biochemical processes. According to the results of biochemical analysis, we could find that the ischemia model was repeated successfully, and Scu and Scue could attenuate neuronal injury.

**Table 1:** Biochemical indicator levels in hippocampal tissue.

Indicator	sham-operated	Model	Nimodipine	Scu	Scue
Mortality (%)	0	12.5%	12.5%	25%	0
Cerebral water (%)	77.39 ± 0.4723	80.82 ± 0.411^△△^	78.74 ± 0.3878**	77.05 ± 0.5934**	76.45 ± 0.4046**
Na^+^ (mmol/L)	53.26 ± 1.753	73.31 ± 3.483^△△^	61.46 ± 8.31**	56.42 ± 4.582**	58.34 ± 3.653**
K^+^ (mmol/L)	83.27 ± 9.498	53.74 ± 6.631^△△^	69.01 ± 4.230*	79.81 ± 4.550**	76.71 ± 4.027**
Na^+^, K^+^-ATPase (μmolpi/mgprot/hour)	10.15 ± 1.936	5.696 ± 1.230^△△^	7.112 ± 1.354**	7.455 ± 0.7323**	7.867 ± 0.6373**
MDA (nmol/mgprot)	6.131 ± 0.826	8.579 ± 1.144^△^	7.431 ± 1.513*	7.842 ± 0.4015**	7.601 ± 0.3442**
SOD (U/mgprot)	147.3 ± 6.395	115.6 ± 7.225^△△^	128.8 ± 3.437**	131.8 ± 4.423**	135.3 ± 5.344**
Ca^2+^ (mmol/L)	13.89 ± 3.647	36.24 ± 6.445^△△^	21.81 ± 2.373**	25.4 ± 4.859**	27.34 ± 3.044**
Ca^2+^-ATPase (μmolpi/mgprot/hour)	7.332 ± 0.3473	4.694 ± 0.3524^△△^	5.457 ± 1.56*	5.675 ± 0.4855*	5.547 ± 1.545*

For statistical analysis: *vs* sham-operated group

^△^ *p* < 0.05

^△△^ *p* < 0.01; *vs* model group

*p < 0.05

**p < 0.01.

### Metabolomic profiling and multivariate data analysis

In the process of sample processing, rapid freeze fixation techniques is very important to adequately preserve the metabolites in vivo levels. Throughout the experiment, sample collection were carried out at low temperature, and quickly placed in the –80°C refrigerator, therefore, the measuring metabolite levels were relatively stable. According the reports, frozen centrifugation and quickly storage at –80°C could adequately preserve the metabolites in vivo levels [19, 30].

Using the optimal chromatography conditions as described, typical base peak intensity (BPI) chromatograms of urine, plasma, and tissue samples were collected in positive and negative modes. Low molecular mass metabolites were well-resolved after 15 min. Subtle changes among these complex data were found using multivariate data analysis techniques, including PCA, OPLS-DA, and PLS-DA.

The data obtained from urine, plasma, and hippocampal tissue samples were analyzed by PCA. A total of 2280 ions in urine, 723 ions in plasma, and 543 ions in hippocampal tissue samples were collected from the sham-operated and model groups using positive or negative modes. In the PCA score analysis, each point represents a single sample, and samples with similar metabolic compositions are clustered together while compositionally different metabolites are dispersed.

**Fig 2:**
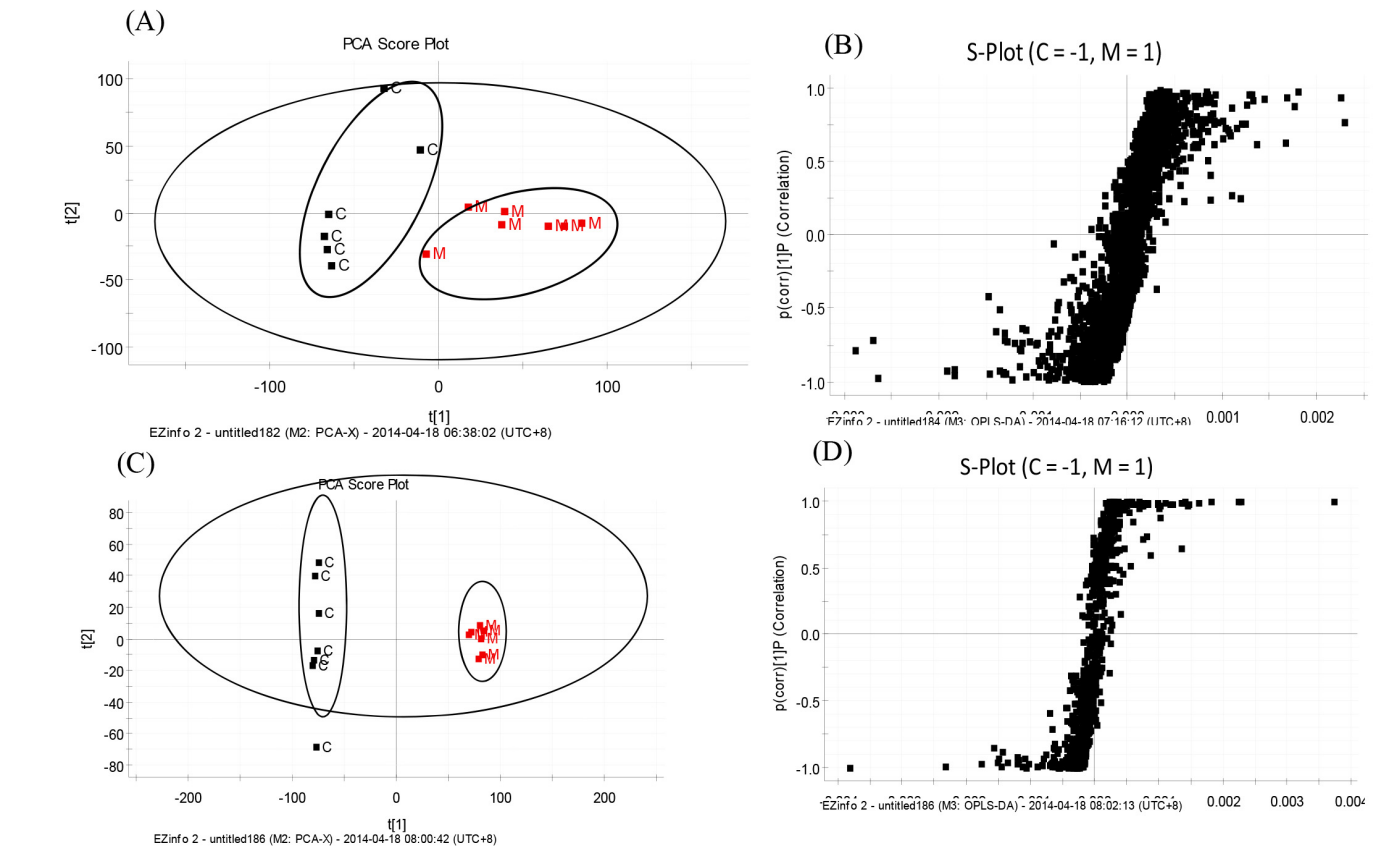
Plasma PCA score plot (A), S-plot of OPLS-DA data (B), hippocampal tissue PCA score plot (C), and S-plot of OPLS-DA data between the sham-operated and model groups in negative mode (D). C, sham-operated group, M, model group.

The plasma samples from the sham-operated and model groups were separated into two clusters in the PCA analysis, indicating that their metabolic profiles had changed as a result of ischemia and reperfusion (Fig 2A and 2B). The PCA score plots provided an overview of the metabolic composition of a group, however, detailed differences between samples in each cluster remained unclear. Significant differences between endogenous metabolites in the sham-operated and model groups were determined from an S-plot of the OPLS-DA. According to the PCA score plot analysis for hippocampal tissue samples, the sham-operated and model groups were separated into two clusters, suggesting that they had different metabolic profiles. The S-plots of OPLS-DA were obtained by MarkerLynx analysis (Fig 2C and 2D).

**Fig 3:**
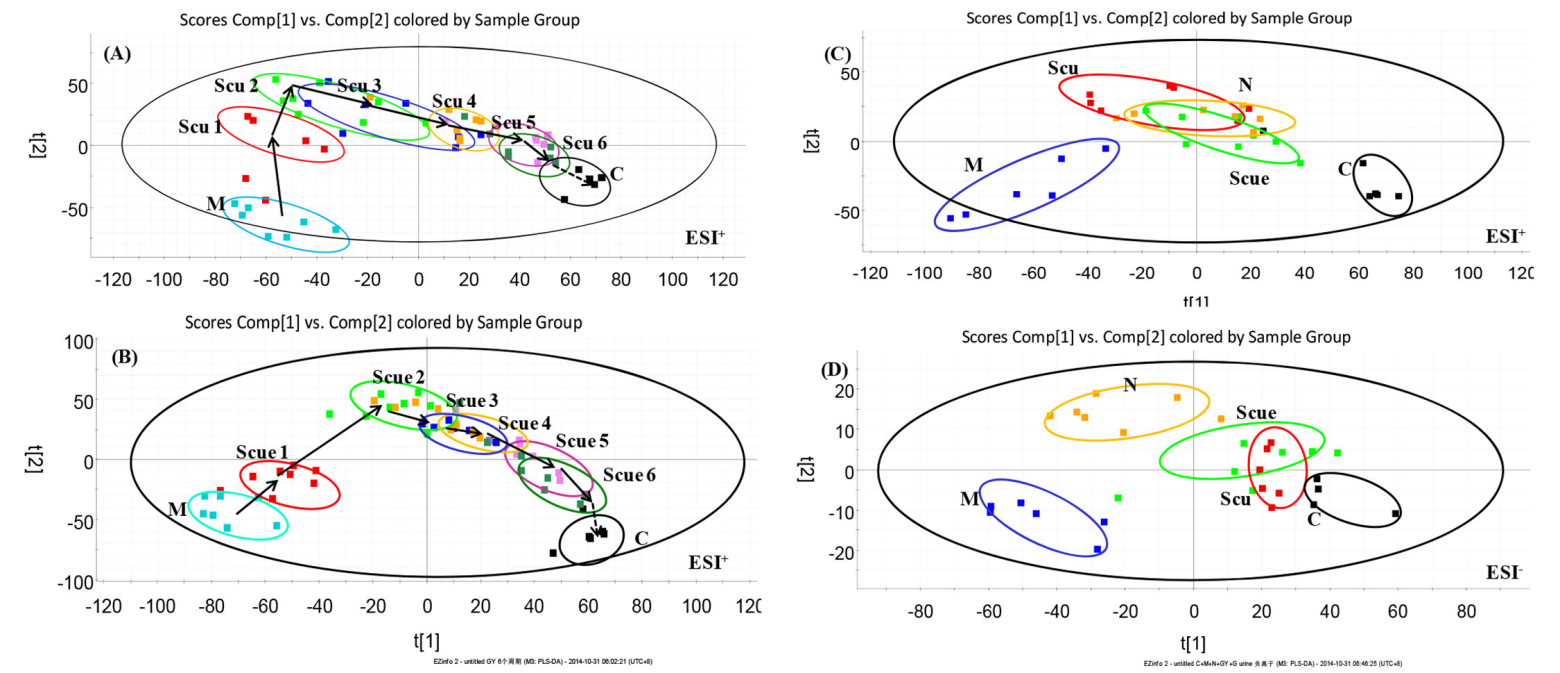
PLS-DA analytical results from urine samples of BCCAO rats treated with Scu (A) or Scue (B) at six therapeutic cycles in positive mode. C, sham-operated group (0–12 h); M, model group (0–12 h); Scu 1–6, after administration of Scu for 0–12 h (Scu1), 12–24 h (Scu2), 24–36 h (Scu3), 36–48 h (Scu4), 48h-60 h (Scu5) and 60–72 h (Scu6); Scue 1–6, after administration of Scue for 0–12 h (Scue1), 12–24 h (Scue2), 24–36 h (Scue3), 36–48 h (Scue4), 48h-60 h (Scue5) and 60–72 h (Scue6). **PLS-DA analytical results from BCCAO rat urine samples treated with Scu, Scue, and nimodipine at 12–24 h in positive (C) and negative modes (D).** C, sham-operated group; M, model group; Scu, after administration of Scu; Scue, after administration of Scue; N, after administration of nimodipine.

To understand the metabolic trajectory of the ischemia model and the protective effects of Scu and Scue, the metabolic pattern of the urine samples was plotted using PLS-DA. The R^2^Y values of the PLS-DA model (Fig 3A and 3B) were 0.871 and 0.84, and Q^2^ was 0.825 and 0.732, respectively, suggesting that the PLS-DA model had goodness of fit and provided valid predictions. Variations in urine metabolic profiling in Scu- and Scue-treated rats were restored to levels similar to those determined in sham-operated rats after treatment. This finding strongly suggested that Scu and Scue had protective effects. Quantitative evaluation of the relative distances between the treatment and sham-operated groups is rarely reported [30, 34]. In the urine samples, relative distances between the treatment and sham-operated groups in the PLS-DA score plot were quantified. The mean metabolic pattern determined in sham-operated rats was used as a reference for the metabolic patterns in treatment groups [30]. We found that the relative distances in untreated ischemic groups decreased after six cycles, and anesthesia effects did not have statistical difference. The relative distances in the Scu- and Scue-treated groups decreased significantly after six therapeutic cycles when compared to the model group, indicating that Scu and Scue may confer protective effects against cerebral ischemic injury (Table 2).

**Table 2:** The relative distance from the PLS-DA scores plot between treatment and sham-operated group urine samples.

		0	1	2	3	4	5	6
Sham- operated	x-axis	67.06	66.32	66.41	65.12	67.22	66.65	66.17
	y-axis	-28.80	-28.91	-27.92	-28.82	-29.32	-27.65	-28.82
Model		132.26±8.91	135.43±10.22	128.32±7.72	117.53±8.04	111.21±9.32	85.67±5.11	59.34±4.72
Scu^a^			130.10±10.81	123.01±8.64	108.61±5.91*	73.06±5.78**	42.01±7.79**	37.45±5.01**
Sham- operated	x-axis	59.56	58.32	59.43	59.17	58.94	57.65	58.47
	y-axis	-61.72	-61.49	-61.05	-60.59	-60.43	-61.28	-61.48
Model		134.81±10.76	138.34±9.21	130.18±7.51	125.53±11.04	112.43±7.36	91.32±9.31	56.66±5.64
Scue^b^			123.34± 10.02	118.15±9.97*	103.87±4.86**	101.59±4.99**	71.16± 6.41**	42.81 ± 6.67**

Table legend

^a^ scutellarin

^b^ scutellarein; compared to the model group

*p < 0.05

**p < 0.01

Urine samples, after administration for 12–24 h, clearly clustered into five groups based on the PLS-DA data, which also provides a comprehensive view of clustering trends for multidimensional data (Fig 3C and 3D). The R^2^Y values of the PLS-DA model were 0.913 and 0.879, in the positive and negative modes, respectively. The Q^2^ values were 0.817 and 0.762, respectively. The relative distances of the urine samples in the PLS-DA score plot are displayed in Table 3. The rats that were pretreated with Scu, Scue, and nimodipine were distributed between the model and sham-operated groups. This suggested that Scu, Scue, and nimodipine could effectively relieve the symptoms of cerebral ischemia by modifying rat metabolic profiles. Furthermore, we found that the Scue group showed metabolite levels that were closer to sham-operated levels when compared to Scu and nimodipine treatment groups, indicating that Scue was more efficacious against cerebral ischemia.

**Table 3:** The relative distance from the PLS-DA scores plot between treatment and sham-operated group urine samples in ESI mode.

	Sham-operated		Model	Scu^a^	N^b^	Scue^c^
ESI mode	x-axis	y-axis				
+	59.45	-27.19	129.72 ± 19.29	105.23 ± 9.49*	72.342 ± 6.21**	58.28 ± 9.23**
-	41.77	-6.66	97.68 ± 9.41	72.15 ± 11.84**	21.26 ± 2.80**	21.36 ± 5.81**

^a^ scutellarin

^b^ nimodipine

^c^ scutellarein; compared to the model group

*p < 0.05

**p < 0.01

### Identification and quantification of potential metabolites

The structural identification of candidate biomarkers was based on the retention time, mass assignment, and online database query. Firstly, the *m/z* values and the precise mass of each metabolite of interest were obtained from loading-plots and VIP-plots constructed following analysis with PLS-DA and OPLS-DA (MarkerLynx), respectively. Secondly, the data for candidate biomarkers were imported into the relevant search databases, such as HMDB and KEGG to preliminarily determine the identities of the metabolites. The mass tolerance between the measured *m/z* values and the exact mass of each candidate metabolite was set to within 10 mDa. Then, the Mass Fragment application manager was applied to facilitate the analysis of the MS/MS spectra of metabolic ions showing a significant difference using the targeted MS/MS mode. Lastly, the metabolites were further identified by comparing their mass spectra and retention behavior with the reference MS/MS spectral data in online databases and the literature [30].

A total of 23 endogenous metabolites were tentatively identified in urine, plasma, and hippocampal tissue samples based on the protocol detailed above. l-Aspartic acid, l-citrulline, and lysoPC (18:0) were identified using standard references, and other endogenous metabolites were tentatively identified according to their retention time and precise molecular mass determined on the UHPLC-MS separation analysis platform. An example is presented below to illustrate the identification process for one endogenous metabolite with a VIP value of 3.02 (t_R_ = 4.15 min, *m/z* 162.1158 in positive ion mode). Its corresponding peak was identified according to its retention time in the total ion chromatogram from an ESI^+^ scan, and an accurate mass of the marker ([M+H]^+^ at *m/z* 162.1158) was determined from the mass spectrum. The MS/MS data regarding the fragmentation pattern of the marker was acquired from the QTOF system. This ion was proposed to contain an odd number of nitrogen atoms because its precise [M + H]^+^ molecular weight was 162.1158, and its molecular formula was speculated to be C_6_H_11_NO_4_ based on the analysis of its elemental composition and fractional isotope abundance. In the positive ion spectrum, the main fragment ions were observed at *m/z* 144.0457, 116.0490, and 70.1201, which corresponded to the loss of–H_2_O,–CO_2_H_2_, and–C_2_O_4_H_4_, respectively from the [M+H]^+^ parent ion. Lastly, this metabolite was tentatively identified as aminoadipic acid using the HMDB database.

Using the protocol described above, 9 endogenous metabolites in urine samples, 8 endogenous metabolites in hippocampal tissue samples, and 6 endogenous metabolites in plasma samples were tentatively identified (Table 4). Some metabolites were significantly upregulated in the model group compared to the sham-operated group, including l-2,3-dihydrodipicolinate, cysteic acid, dihydroceramide, and aminoadipic acid in urine, acetylcarnosine, l-aspartic acid, 4-hydroxyphenylacetaldehyde, and lysoPC(P-18:0) in hippocampal tissue, and cer(d18:0/18:0), *n*-alpha-acetyllysine, and ceramide (d18:1/12:0) in plasma (*p* < 0.05). The significantly downregulated metabolites were deoxycytidine, pyrrolidonecarboxylic acid, isocitric acid, oxalosuccinic acid, and 2-phenylethanol glucuronid in urine, d-pantothenoyl-l-cysteine, citrulline, l-2-hydroxyglutaric acid, and neuraminic acid in hippocampal tissue, and ceramide (d18:1/18:0), *N*-acetylhistamine, and galactinol in theplasma (*p* < 0.05). These changes in endogenous metabolite levels in urine, hippocampal tissue, and plasma may contribute to the differences between cerebral ischemia and normal rats.

**Table 4:** UPLC-MS identification of potential biomarkers.

No	VIP	t_R_(min)	ESI mode	m/z measured	Mass accuracy (ppm)	Formula	Metabolite
Um1^a^	3.13	7.04	[M+H]^+^	170.0375	3.53	C_7_H_7_NO_4_	l-2,3-Dihydrodipicolinate
Um2	7.36	12.89	[M+H]^+^	330.5179	6.35	C_19_H_39_NO_3_	Dihydroceramide
Um3	3.85	0.85	[M+H]^+^	170.0045	7.65	C_3_H_7_NO_5_S	Cysteic acid
Um4	3.02	4.15	[M+H]^+^	162.1558	3.08	C_6_H_11_NO_4_	Aminoadipic acid
Um5	2.59	5.81	[M+H]^+^	130.0932	-9.99	C_5_H_7_NO_3_	Pyrrolidonecarboxylic acid
Um6	3.18	1.00	[M+H]^+^	228.2172	11.83	C_9_H_13_N_3_O_4_	Deoxycytidine
Um7	6.06	1.00	[M-H]^-^	191.1235	-0.52	C_6_H_8_O_7_	Isocitric acid
Um8	4.93	3.08	[M-H]^-^	187.9865	5.32	C_6_H_6_O_7_	Oxalosuccinic acid
Um9	4.44	7.41	[M-H]^-^	297.0998	3.70	C_14_H_18_O_7_	2-Phenylethanol glucuronide
Bm1^b^	3.69	2.58	[M-H]^-^	321.3780	8.09	C_12_H_22_N_2_O_6_S	D-Pantothenoyl-L-cysteine
Bm2	2.92	1.40	[M-H]^-^	267.1439	4.12	C_11_H_16_N_4_O_4_	Acetylcarnosine
Bm3	2.54	1.02	[M-H]^-^	174.1857	9.76	C_6_H_13_N_3_O_3_	Citrulline
Bm4	1.11	0.84	[M-H]^-^	132.1027	-5.30	C_4_H_7_NO_4_	l-Aspartic acid
Bm5	5.34	1.03	[M+H]^+^	137.0736	8.02	C_8_H_8_O_2_	4-Hydroxyphenylacetaldehyde
Bm6	1.51	4.58	[M+H]^+^	149.0524	-8.05	C_5_H_8_O_5_	L-2-Hydroxyglutaric acid
Bm7	1.84	4.02	[M+H]^+^	508.3998	0.79	C_26_H_54_NO_6_P	LysoPC(P-18:0)
Bm8	4.35	1.02	[M+H]^+^	268.1513	-2.98	C_9_H_17_NO_8_	Neuraminic acid
Pm1^c^	2.45	7.75	[M-H]^-^	564.5660	3.01	C_36_H_71_NO_3_	Ceramide (d18:1/18:0)
Pm2	3.21	8.69	[M-H]^-^	566.5862	-1.59	C_36_H_73_NO_3_	Cer(d18:0/18:0)
Pm3	1.73	2.82	[M-H]^-^	187.0910	-1.07	C_8_H_16_N_2_O_3_	N-Alpha-acetyllysine
Pm4	1.79	7.64	[M-H]^-^	482.4558	-3.73	C_30_H_59_NO_3_	Ceramide (d18:1/12:0)
Pm5	1.02	3.21	[M-H]^-^	152.0992	0.66	C_7_H_11_N_3_O	N-Acetylhistamine
Pm6	2.08	3.21	[M+H]^+^	343.0788	9.91	C_12_H_22_O_11_	Galactinol

^a^ urine metabolite

^b^ brain metabolite

^c^ plasma metabolite.

**Fig 4:**
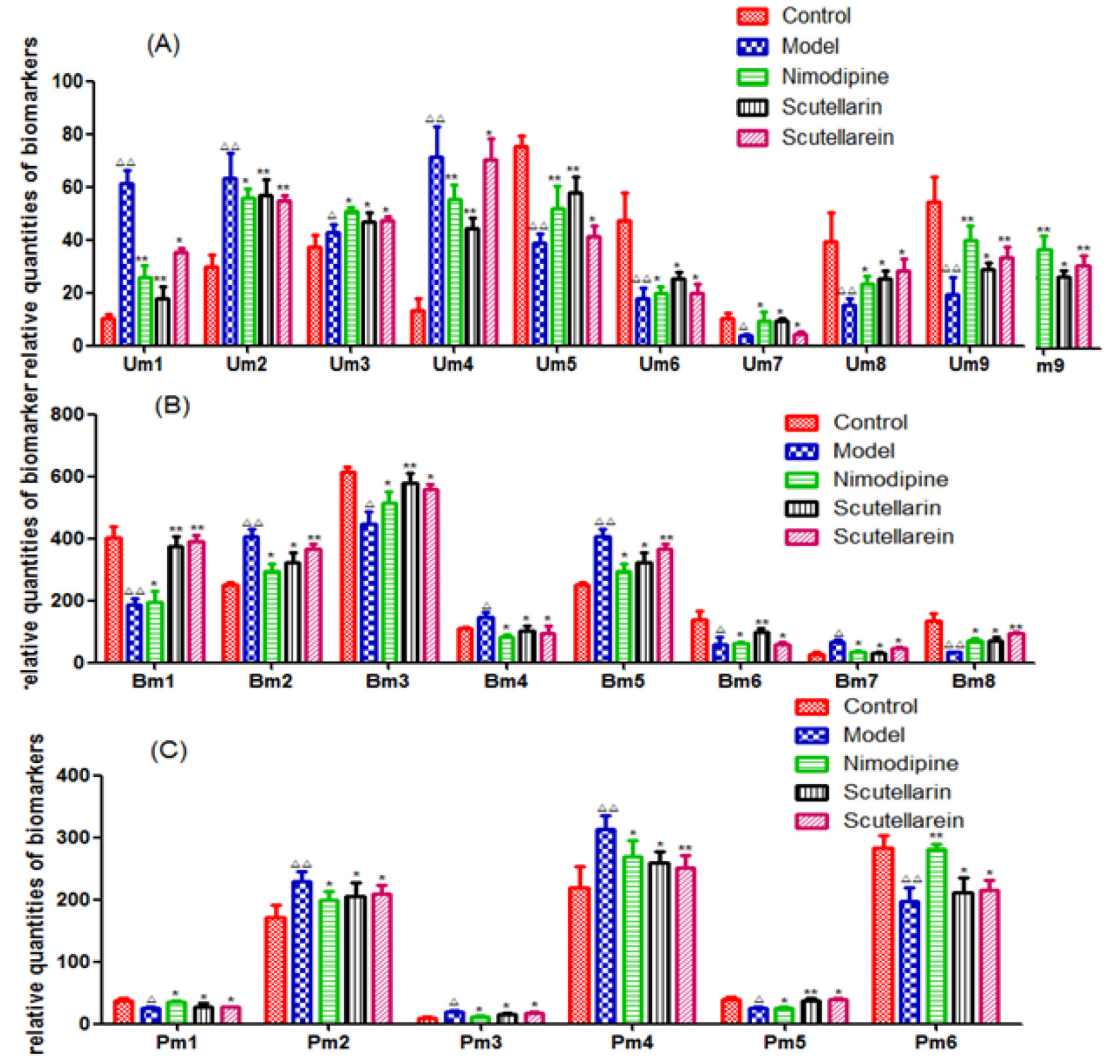
Changes in the relative quantities of target metabolites identified in different groups. Um, urine metabolites; Bm, brain metabolites; and Pm, plasma metabolites. Statistical analysis compared with the sham-operated group, ^△^ *p* <0.05, ^△△^ *p*<0.01, and compared with the model group, *p<0.05, **p<0.01; (A) 9 endogenous metabolites in the urine; (B) 8 endogenous metabolites in the brain tissue; and (C) 6 endogenous metabolites in the plasma.

Additionally, changes in the relative quantities of the endogenous metabolites identified in different groups were analyzed to characterize the protective effects of Scu and Scue (Fig 4). The quantities of these key biomarkers were more similar to those of the Sham-operated group when compared to the model group. Furthermore, the relative quantities of these biomarkers were altered after pretreatment with Scu and Scue, according to a parametric *t*-test. This result indicated that Scu and Scue might regulate endogenous metabolite levels.

### Metabolic pathway and function analysis

**Fig 5:**
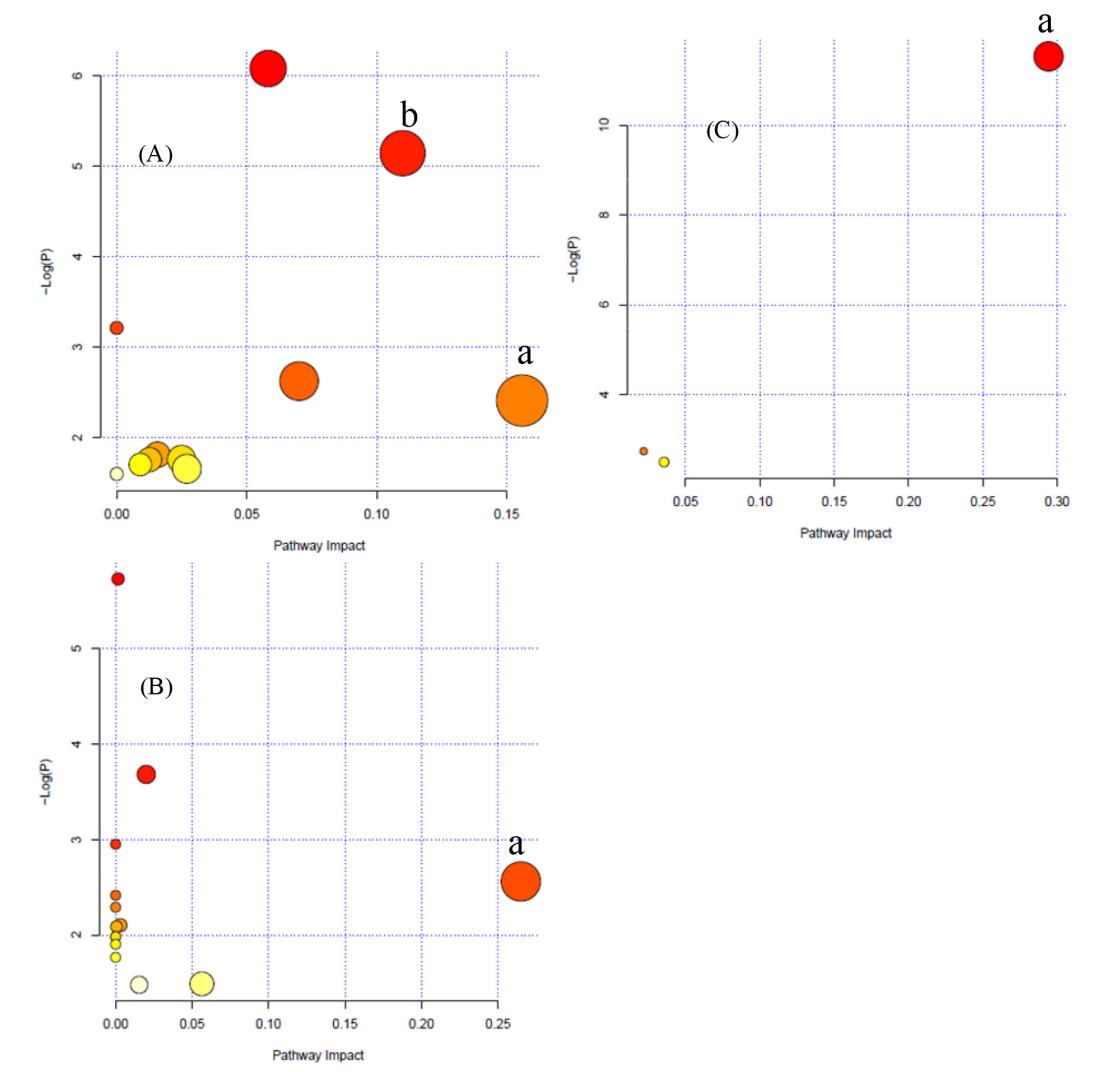
Metabolomics pathway analysis (MetPA) summary. (A) Urine, a) sphingolipid metabolism and b) lysine biosynthesis; (B) hippocampal tissue, a) alanine, aspartate, and glutamate metabolism; and (C) Plasma, a) sphingolipid metabolism.

To determine possible pathways contributing to ischemia-reperfusion injury, a metabolomics pathway analysis (MetPA) was performed to identify metabolic pathways and their networks using an online database. This analysis resulted in the construction of 11 metabolic pathways in urine, 14 metabolic pathways in hippocampual tissue, and 3 metabolic pathways in the plasma (Fig 5) that were important for host-responses to ischemic injury. Among the metabolic pathways identified, sphingolipid metabolism (impact-value: 0.16) and lysine biosynthesis (impact-value: 0.11) in urine; alanine, aspartate, and glutamate metabolism (impact-value: 0.26) in hippocampal tissue; and sphingolipid metabolism (impact-value: 0.29) in plasma were determined to be the most important.

Previous studies determined that cerebrovascular disease patients have prevalent neurodegenerative disorders, such as age-related learning and memory deficits. By using an untargeted metabolomics study, sphingomyelin-ceramide metabolism was shown to be disturbed, and relevant endogenous metabolites, such as *N*,*N*-dimethylsphingosine, were upregulated in the dorsal horns of rats with neuropathic pain [36]. The sphingolipid family includes bioactive molecules that not only serve as integral components of cellular membranes, but they also play critical roles in signaling and other cellular processes [37]. These discoveries supported our results regarding sphingolipid metabolism and altered endogenous levels of the dihydroceramide and ceramide metabolites. In our study, significant increases in dihydroceramide and ceramide levels were observed in the model group, while their levels decreased in the Scu- and Scue-treated groups. Dihydroceramide and ceramide are classified as sphingolipids, and they play important roles in sphingolipid metabolism [38–40]. Accumulating evidence indicates that ceramide is closely involved in apoptosis in neurodegenerative disorders and during aging, and dihydroceramide, the ceramide precursor, accumulates in hypoxic conditions [41–43]. Based on these findings, we believe that ceramide regulation plays an important role in neurological disease.

Excessive release of amino acid neurotransmitters is regarded as an important pathogenesis mechanism during ischemia-reperfusion [44]. This depolarization is mediated by metabolic failure caused by ischemia, and results in the influx of Ca^2+^ via voltage-sensitive Ca^2+^ channels. This leads to a concomitant flood of amino acid neurotransmitters, especially glutamate and aspartic acid [45], that are released into the synaptic cleft. Then, the excitatory amino acid neurotransmitters induce a cascade of events leading to cell death [46, 47]. In our study, two unique amino acid metabolic pathways, alanine, aspartate, and glutamate metabolism as well as lysine biosynthesis, were identified in our ischemia model. Furthermore, l-aspartic acid was identified as a potential biomarker. l-aspartic acid is a nonessential amino acid that is made from glutamic acid by enzymes that utilize vitamin B6. It has important roles in alanine, aspartate, and glutamate metabolism, and it is a major excitatory neurotransmitter that is increased in epileptic and stroke patients. Additionally, the relative metabolite intensities of various amino acids, such as 1-pyrroline-2-carboxylic acid, aminoadipic acid, *N*-acetylhistamine, l-2,3-dihydrodipicolinate, cysteic acid, and pyrrolidonecarboxylic acid, were up- or downregulated in the model group, indicating that the metabolism of amino acids was altered during ischemia and hypoxia. Aminoadipic acid is a metabolite in the principal lysine biochemical pathway. It antagonizes neuroexcitatory activity modulated by the glutamate receptor, *N*-methyl-d-aspartate. Taurine is an important inhibitory amino acid, and it has also been reported that taurine decreased the effects of cerebral ischemia injury by counteracting the toxicity of excitatory amino acids [48]. Cysteic acid is a crystalline amino acid formed via the oxidation of cysteine, and it is a precursor of taurine. The relative intensity of cysteic acid was upregulated in the model group. After pretreatment with scutellarin, scutellarein, and nimodipine, the relative intensity of cysteic acid was further upregulated, indicating that the concentration of taurine increased and thus contributed to the protection of nerve cells from damage.

### Analysis of the correlation between biomarkers and biochemical indicators

The results of correlation analysis between potential biomarkers and biochemical indicators in hippocampal tissue are presented in Table 5. Na^+^ levels had a strong positive association with metabolites Bm2 (brain tissue metabolites) (acetylcarnosine) (r = 0.418) and Bm7 (lysoPC(P-18:0)) (r = 0.748) and a negative association with metabolites Bm1 (*D*-pantothenoyl-*L*-cysteine), Bm3 (citrulline), and Bm8 (neuraminic acid) (r < –0.405). However, K^+^ only showed a significant association with Bm8 (r = 0.543). Furthermore, the level of Na^+^/K^+^-ATPase was significantly and positively associated with Bm1, Bm4 (l-aspartic acid), and Bm8 (r > 0.543), and it was negatively associated with Bm2, Bm6, (*L*-2-hydroxyglutaric acid) and Bm7 (r < –0.457). A positive association was discovered between Ca^2+^ levels and Bm2, Bm4, Bm5 (4-hydroxyphenylacetaldehyde), and Bm7 (r > 0.465), while levels were negatively associated with Bm1 and Bm6 (r < –0.520). Ca^2+^-ATPase was only associated with Bm1 (r = 0.600). Meanwhile, the level of MDA showed a highly negative correlation with metabolites Bm1, Bm3, and Bm8 (r < –0.434), and the level of SOD was significantly and positively associated with Bm2, Bm5, and Bm8 (r > 0.474) and negatively associated with Bm3 (r = –0.549).

**Table 5:** Correlative analysis of biomarkers and biochemical data in hippocampal tissue.

Indicator	Bm^a^1	Bm2	Bm3	Bm4	Bm5	Bm6	Bm7	Bm8
Na^+^	–0.444*	0.418*	–0.578**	0.233	0.418*	–0.405	0.748**	–0.599**
K^+^	0.355	–0.366	0.386	–0.202	–0.366	0.117	–0.281	0.543*
Na^+^,K^+^-ATPase	0.564**	–0.457*	–0.213	0.543*	0.370	–0.457*	–0.564**	0.872**
Ca^2+^-ATPase	0.603**	–0.295	0.023	–0.376	–0.295	0.182	–0.388	0.342
Ca^2+^	–0.600**	0.465*	–0.387	0.522*	0.465*	–0.520*	0.708**	–0.217
MDA	–0.434*	0.320	–0.532*	0.283	0.320	0.080	0.374	–0.577**
SOD	–0.429	0.474*	–0.549**	–0.189	0.689**	0.137	–0.348	0.474*

^a^ brain metabolite

*p < 0.05

**p < 0.01

The results of the correlation analysis demonstrated that the endogenous metabolites levels and biochemical indicators correlated well in hippocampal tissue. The levels of Na^+^/K^+^-ATPase and Ca^2+^ were closely related to the metabolite l-aspartic acid. It has been reported that l-aspartic acid, as an important excitatory amino acid, can induce intracellular Ca^2+^ overload, leading to cell death during cerebral ischemic injury. Correlative analyses can be used to establish relationships between biomarkers and biochemical indicators. More importantly, these promising biomarkers can provide guidance for the clinical diagnosis and therapy after ischemic stroke.

## Conclusion

In this study, we profiled metabolic features and constructed metabolite networks to analyze the neuroprotective effect of Scu- and Scue-treatment of neural injury induced by cerebral ischemia-reperfusion. Metabolomic trajectories and pathways active in ischemic injury were determined through UHPLC-QTOF/MS coupled with multivariate statistical analysis. The protective effects of Scu and Scue were evaluated for their ability to regulate pathways disrupted by ischemic injury. A total of 23 endogenous metabolites as well as 11 metabolic pathways in urine, 14 metabolic pathways in hippocampal tissue, and 3 metabolic pathways in the plasma were identified. Sphingolipid metabolism, lysine biosynthesis, and alanine, aspartate, and glutamate metabolism were determined to be the most important metabolic pathways. The metabolic deviations occurring after ischemic injury were nearly recovered to sham-operated levels after Scu or Scue intervention. Additionally, Scue exhibited heightened protective effects compared to Scu, as determined by metabolic deviations that recovered more closely to sham-operated levels, according to relative distance calculations. These results suggested that Scu and Scue might play critical roles in the treatment of ischemic injury through up- and downregulation of endogenous metabolite levels. These findings will facilitate the development of clinical applications of Scu and Scue and further new drug development of Scue and its derivatives.
